# Brief Report: The Four Most Frequently Diagnosed Vector-borne Diseases Among Service Member and Non-Service Member Beneficiaries in the Geographic Combatant Commands, 2010–2022

**Published:** 2024-01-20

**Authors:** Ralph A. Stidham, Ronald Cole, Sithembile L. Mabila

**Affiliations:** 1 Epidemiology and Disease Surveillance Department, U.S. Army Public Health Command–West, Joint Base San Antonio–Fort Sam Houston, TX; 2 Human Health Services, U.S. Public Health Command–Pacific, Tripler, HI; 3 Epidemiology and Analysis Branch, Armed Forces Health Surveillance Division, Defense Health Agency, Silver Spring, MD

## 1 BACKGROUND

Vector-borne diseases (VBDs) may pose an increased risk for U.S. service members during recurring military training exercises, operations, and response missions, in addition to residence in endemic regions within and outside the continental U.S. [1,2] Prior *MSMR* reports address VBD surveillance, described by surveillance data for 23 reportable medical events (RMEs), among active duty and reserve component service members. [3,4] This report covers a 13-year surveillance period, from January 2010 to December 2022, and provides linear trends of selected VBDs among Armed Forces service and non-service member beneficiaries diagnosed at installations within the Northern Command (NORTHCOM), Africa Command (AFRICOM), Central Command (CENTCOM), European Command (EUCOM), Indo-Pacific Command (INDOPACOM), or Southern Command (SOUTHCOM). Trends of only the 4 most-frequently reported VBDs were evaluated, as Lyme disease, malaria, Rocky Mountain Spotted Fever (RMSF), and dengue fever comprised 90% (n=5,199) of all 23 VBDs (n=5,750) among Military Health System (MHS) beneficiaries documented as RMEs during the surveillance period.

## 2 METHODS

This study includes all MHS beneficiaries from January 2010 through December 2022. Data were acquired from RME records of 23 VBDs from the Defense Medical Surveillance System (DMSS), limited to the 4 most-diagnosed VBDs in DMSS during the surveillance period; a full listing of VBD RMEs are available in a prior *MSMR* report. [3] A VBD case was defined as an individual identified through a RME report, classified as “confirmed,” “probable,” or “suspect” by having met specified laboratory or epidemiologic criteria. [6]

Demographic information including military component (active, reserve, guard), beneficiary status (service members or non-service member), and U.S. Combatant Command (CCMD) at time of diagnoses were included. Non-service member beneficiaries included dependents, former service members, and retirees. MHS beneficiaries diagnosed as a case before the surveillance period were excluded. An individual could qualify as a case once for each RME type. Incidence date was the earliest event date, with classification determined by utilizing all available data, prioritizing confirmed over probable or suspect records.

## 3 RESULTS

A total of 5,199 confirmed, probable, and suspect cases of Lyme disease (n=3,400), RMSF (n=893), malaria (n=679), and dengue fever (n=227) were identified among MHS beneficiaries from January 2010 through December 31, 2022 (**Table 1**). Of those confirmed, probable, and suspect cases, 2,343 were diagnosed in service members and 2,918 were diagnosed in non-service member beneficiaries (data not shown). Lyme disease and RMSF, both caused by tick-borne pathogens, accounted for 83% of cases, while malaria and dengue fever, transmitted by mosquito vectors, comprised the remainder.

**Figure (1):**
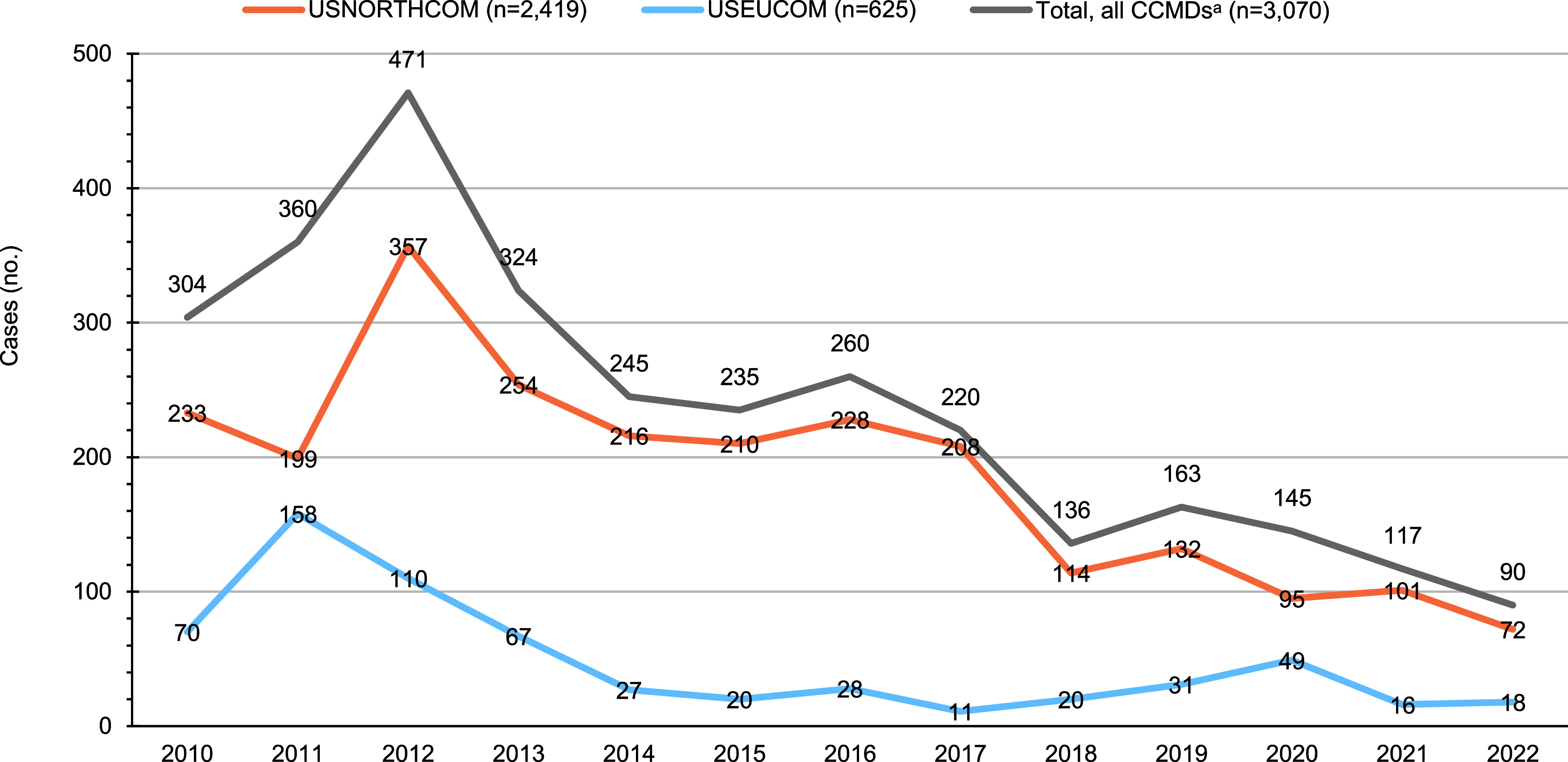
Confirmed and Probable Lyme Disease Cases by Selected U.S. Combatant Commands for MHS Service and Non-Service Member Beneficaries, 2010–2022

Since Lyme disease was the most common VBD of the 4 diseases evaluated during the surveillance period, trends of confirmed and probable cases of Lyme disease over time by CCMD are presented in the **Figure 1**. Confirmed Lyme disease cases peaked in 2012 (n=455) and then gradually decreased over the study period to a low of 75 cases in 2022; probable cases peaked in 2017 (n=53) and steadily decreased to a low of 15 cases in 2022; suspect cases peaked in 2016 (n=73) and progressively declined to a low of 8 cases in 2022 (data not shown). Cases from NORTHCOM represented the greatest number of confirmed and probable Lyme disease cases during the entire surveillance period (**Figure 1**). The annual number of confirmed and probable Lyme disease cases from EUCOM were greatest in 2011 and lowest in 2017; Lyme cases were very low in all other CCMDs, ranging from 0 to 6 cases annually (data not shown). The Atlantic and Central regions of the U.S contributed 85% of NORTHCOM’s reported RMSF cases (data not shown). NORTHCOM averaged 30 RMSF cases annually between 2010 and 2016, dramatically increasing to an average of 149 cases between 2017 and 2019 (data not shown). NORTHCOM was only able to confirm 32% of RMSF cases reported during the surveillance period (**Table 1**).

## 4 DISCUSSION

Lyme disease cases constituted the largest proportion of overall RMEs in this report, with highest numbers occurring in 2012. A substantial proportion of Lyme disease cases were reported from locations in the northeastern U.S., where Lyme disease is known to be endemic: 43% of service members and non-service beneficiaries were diagnosed at NORTHCOM Groton (New London Submarine Base, CT) and NORTHCOM New England. The New London Submarine Base is close to Lyme, Connecticut, where an epidemiological evaluation of a cluster of children with arthritis resulted in the first complete description of the infection in 1976, giving the disease its name. [8] Connecticut still ranks in the top 10 states for reported Lyme disease cases. [9] No Lyme disease cases were reported in AFRICOM during the surveillance period, because the vectors (*Ixodes scapularis* and *Ixodes scapularis*) are not present in the region.

In 2017, the Armed Forces expanded its RME guidelines to include all spotted fever rickettsioses (SFR), to better align with CDC case definitions. [2] Diagnoses and reports of rickettsial diseases at military hospitals and clinics in NORTHCOM (where RMSF is endemic) significantly increased after the surveillance requirement expansion from only RMSF to the broader SFR group. In this review, all SFR cases were RMSF diagnoses (n=893).

Approximately 68% of RMSF cases reported during the surveillance period could not be confirmed. All laboratory tests performed at military health facilities for RMSF were Indirect Fluorescent Antibody (IFA) assay and other antibody tests, and no records of testing with PCR of blood or eschar specimens were found. Definite identification of *Rickettsiae* is not feasible solely by IFA due to considerable serologic cross-reactivity, particularly when high-endpoint titers are seen for more than 1 rickettsial antigen. [10] Increased use of molecular assays (i.e., real-time PCR) can both confirm and offer species-specific diagnosis in a single sample, facilitating identification and management of rickettsial diseases in both service members and non-service beneficiaries.

The observed decline in the incidence of mosquito-borne cases, such as malaria and dengue, among deployed service members over the last decade is likely due to reduced deployments to endemic regions, with the exception of EUCOM. [4] Although dengue fever is not represented significantly in EUCOM in this study, there is a rising risk of dengue and other VBDs due to environmental changes and expanding global travel and trade. [11,12,13]

VBDs often manifest with non-specific symptoms, and when unconfirmed could constitute a number of other infections or health conditions. Lyme disease is frequently misdiagnosed as chronic fatigue syndrome, fibromyalgia, or multiple sclerosis. This non-specificity of symptoms and related issues such as diagnostic availability and cross-reactivity in diagnosis confirmation can pose challenges for accurate case identification and classification, resulting in the major limitations to this study’s findings.

This report summarizes data from electronic reports of RMEs and examines the incidence and geographic distribution of the top 4 vector-borne infectious diseases among service members and non-service MHS beneficiaries in the CCMDs during a recent 13-year period. Awareness of the risk of these VBDs will help senior leaders develop and employ strategies to decrease avertable medical problems in MHS beneficiaries, maximizing the productivity and readiness of the medical force.

## Figures and Tables

**Table (1):** Four Most Frequently Reported Vector-borne Disease Cases^a^ by U.S. Combatant Command Region and Case Classification, MHS Service and Non-Service Member Beneficiaries, 2010–2022

**Disease and Case Classification**	**No.**	**USNORTHCOM**	**USAFRICOM**	**USCENTCOM**	**USEUCOM**	**USINDOPACOM**	**USSOUTHCOM**	**Total**
		**No.**	**No.**	**No.**	**No.**	**No.**	**No.**	**No.**
**Lyme disease**	2721	0	4	648	26	1	3400
Confirmed	2132	0	4	576	18	1	2731
Probable	287	0	0	49	3	0	339
Suspect	302	0	0	23	5	0	330
**Malaria**	457	16	92	69	45	0	679
Confirmed	436	14	88	65	43	0	646
Probable	6	0	0	1	0	0	7
Suspect	15	2	4	3	2	0	26
**Rocky Mountain Spotted Fever**	885	0	0	3	5	0	893
Confirmed	279	0	0	1	2	0	282
Probable	454	0	0	0	1	0	455
Suspect	152	0	0	2	2	0	156
**Dengue Fever**	165	16	1	6	24	15	227
Confirmed	134	10	1	5	19	15	184
Probable	20	4	0	1	1	0	26
Suspect	11	2	0	0	4	0	17

Abbreviations: MHS, Military Health System; USNORTHCOM, U.S. Northern Command; USAFRICOM, U.S. Africa Command; USCENTCOM, U.S. Central Command; USEUCOM, U.S. European Command; USINDOPACOM, U.S. Indo-Pacific Command; USSOUTHCOM, U.S. Southern CommandThe data source for all record types was the DMSS with the exception of laboratory data, which were from NMCPHC.

^a^Four most-frequently reported vector-borne disease cases among Lyme disease, malaria, Rocky Mountain Spotted Fever, Dengue fever, Zika virus infection, Chikungunya, arboviral diseases, ehrlichiosis/anaplasmosis, leishmaniasis, trypanosomiasis, tularemia, relapsing fever, typhus, Rift Valley fever, babesiosis, hemorrhagic fevers, other mosquito-borne viral fever, bartonellosis, filariasis, plague, yellow fever, Japanese encephalitis, and sandfly fever.
